# Complete Genome Sequence of Dengue Virus Serotype 3 from Guangzhou, China

**DOI:** 10.1128/genomeA.00208-12

**Published:** 2013-03-07

**Authors:** Zhijun Bai, Li-Cheng Liu, Li-Yun Jiang, Qi Liu, Yi-Min Cao, Yang Xu, Qin-Long Jing, Lei Luo, Zhi-Cong Yang, Yong-Qiang Jiang, Weijun Chen, Biao Di

**Affiliations:** a Guangzhou Center for Disease Control and Prevention, Guangzhou, Guangdong, China; b State Key Laboratory of Pathogen and Biosecurity, Institute of Microbiology and Epidemiology, Academy of Military Medical Sciences, Beijing, China; c BGI in Shenzhen, Shenzhen, China

## Abstract

In 2009, dengue virus serotype 3 (DENV-3) was first detected in Guangzhou, China. In this study, we identified another isolated strain belonging to genotype II. Phylogenetic analysis shows that the GZ/10476/2012 strain has a close relationship with the DENV-3 genotype II from Southeast Asian strains.

## GENOME ANNOUNCEMENT

Dengue virus (DENV) is a mosquito-borne virus which causes a wide range of human diseases, from the acute febrile illness dengue fever (DF) to life-threatening dengue hemorrhagic fever/dengue shock syndrome (DHF/DSS). It is estimated that the annual worldwide occurrences of DF and DHF are, respectively, 100 million and 250,000 cases, and the case mortality number is 25,000. Dengue virus (DENV) has four serologically distinct serotypes (DENV-1, DENV-2, DENV-3, and DENV-4) (1, 2). All the DENV serotypes are further classified into multiple subtypes/genotypes based on their genomic diversity (3). Five distinct genotypes of DENV-3 have been identified to date (4, 5). Genotypes I to III are responsible for most DENV-3 infections and have been associated with DF/DHF epidemics in Southeast Asia, the Indian subcontinent, the south Pacific, East Africa, and the Americas. Genotypes IV and V have not been not associated with DHF epidemics and are represented by only a few early sequences from the Americas, the south Pacific, and Asia, with the exception of recent reports regarding genotype V-related cases from Brazil and Colombia (6).

DENV-3 has occurred in many provinces of southern China, and Guangdong province is the major affected area in China. DENV-3 genotypes II, III, and V were identified in Guangzhou, China (7). In this study, another DENV-3 genotype was investigated.

A dengue virus serotype 3 (DENV-3) was isolated from a 33-year-old female DF patient on 3 August 2012, 2 days after the onset of symptoms. The complete genome was determined by PCR and sequencing. The length of this isolated virus is 10,630 bp. Phylogenetic analyses based on the nucleic acid sequence of the complete envelope gene and genome were conducted using the neighbor-joining method in MEGA 5.04 software. The result shows that the GZ/10476/2012 strain has a close relationship with DENV-3 genotype II from Southeast Asian strains, including 98TW407 from Taiwan and DENV-3/TH/BID-V2315/2001 from Thailand. Compared with those strains, the amino acid homologies of this strain are 99.3% and 98.9%, respectively. However, other DENV-3 strains (EU367962, GU363549, and AF317645) isolated from China have been reported, and these belonged to genotypes II, III, and V, respectively. Compared to those strains, the amino acid homologies of the GZ/10476/2012 strain are 99.0%, 97.7%, and 97.9%, respectively. Those results indicated that the GZ/10476/2012 strain may be a novel linage circulating in Guangzhou.

This outbreak of DF in Guangzhou in 2012 was possibly initiated by the introduction of DENV-3 from Southeast Asia. It is difficult to determine the route of DENV-3 imported in China because the data are limited. But one could speculate that the introduction of DENV-3 into Guangzhou occurred in 2009, because DENV-3 had not been detected in that area before 2009. More studies are needed to confirm the origin of dengue virus 3 in Guangzhou.

### Nucleotide sequence accession number.

The virus genome sequence described here has been deposited in the GenBank database and assigned accession no. KC261634.
